# Relationship between nursing students’ levels of liking children and self-efficacy in paediatric medication administration

**DOI:** 10.1186/s12909-024-06386-y

**Published:** 2024-11-27

**Authors:** Mehmet Bulduk, Veysel Can, Eda Nur Muhafiz

**Affiliations:** 1 Faculty of Health Sciences, Department of Nursing, Van Yüzüncü Yıl University, Van, Türkiye, 65000, +905304333476 Turkey; 2 Agri Provincial Directorate of Health, Agri Taşliçay State Hospital, Agri, 04000 Turkey

**Keywords:** Child, Drug preparation, Drug administration, Nursing students, Self-efficacy

## Abstract

**Background:**

Medication management is a critical aspect of nursing, in particular with respect to paediatric patients, in whom medication errors are prevalent. Enhancing competence in this area requires not only general nursing skills but also targeted educational interventions and emotional support. This study aims to examine the relationship between nursing students’ levels of liking children and their self-efficacy in paediatric medication administration, thereby exploring the impact of emotional factors on clinical competence.

**Methods:**

This descriptive study was conducted with 308 nursing students in their second, third and fourth years of study at a state university in eastern Turkey between December 2022 and February 2023. Data were collected using the Student Descriptive Information Form, the Barnett Liking of Children Scale and the Medication Administration Self-Efficacy Scale. Statistical analysis included the Mann–Whitney U test, the Kruskal–Wallis H test, Dunn’s test for multiple comparisons and Spearman’s rho correlation coefficient.

**Results:**

The mean age of the participants was 21.82 ± 1.94 years, and 68.8% were female. The mean Medication Administration Self-Efficacy Scale score was 52.97 ± 15.27, and the mean Liking of Children Scale score was 66.65 ± 15.8. No significant relationship was found between the total score on the Liking of Children Scale and the score on the drug preparation subdimension (*p* > 0.05). However, a positive but weak correlation was found between the total score on the Liking of Children Scale and the score on the drug administration subdimension (*r* = 0.137; *p* < 0.05). Similarly, a positive but weak relationship was identified between the total score on the Liking of Children Scale and the score on the Medication Administration Self-Efficacy Scale (*r* = 0.123; *p* < 0.05).

**Conclusions:**

Nursing students’ liking of children is positively, although weakly, associated with their self-efficacy in paediatric medication administration. This result suggests that emotional factors, such as affinity for children, may be relevant when designing nursing education programmes, particularly in paediatric care settings .

## Background

The paediatric population is more vulnerable to medication errors than adults are. Studies have shown that medication errors are approximately eight times more common in paediatric patients than in adults, and these errors can lead to severe consequences because of physiological factors [1]. In paediatric intensive care units, errors related to improper dosing and timing are common and pose significant threats to children’s health [2–4]. Therefore, the careful management of medication administration in paediatric clinics is crucial for patient safety [5].

For nursing students, practising in paediatric clinics is often a stressful and anxiety-inducing experience [6, 7]. The diverse developmental stages of children and the need for family-centred care can negatively affect students’ clinical performance, increasing the risk of medication errors. Self-efficacy plays a critical role in helping students cope with these challenges [8, 9]. Students with high self-efficacy experience less stress and anxiety during clinical practice and demonstrate confidence in their clinical skills [10, 11].

The affection that nursing students feel towards children may positively influence their self-efficacy. Studies have shown that students who empathise with children are more successful in medication administration and are less prone to making errors than are students who do not [12]. No specific studies in the literature have examined the relationship between nursing students’ affection for children and their self-efficacy in paediatric medication administration. The varying levels of clinical experience among the nursing students included in this study provide a valuable opportunity to observe how experience influences the development of certain skills. Second-year students, although they have not yet taken the child health course, encounter paediatric patients in clinics such as ear, nose, and throat (ENT), orthopaedics, and neurosurgery. Third-year students have completed the child health course during the fall semester, gaining foundational knowledge in paediatrics, whereas fourth-year students represent the group with the highest level of clinical experience. In light of these different experience levels, this study aims to explore the relationship between nursing students’ affection for children and their self-efficacy in paediatric medication administration.

## Methods

### Type of research

This study used a descriptive and correlational design.

### Place and time of research

The study was conducted in the nursing department of a state university located in eastern Turkey between February 2022 and February 2023. No sampling method was used, and efforts were made to reach all second-, third- and fourth-year nursing students in the population. The inclusion and exclusion criteria were as follows:

### Inclusion criteria

- Second-, third- and fourth-year students with clinical experience and developing medication administration skills.
- Students who attended classes on days data were collected and who voluntarily agreed to participate in the study.

### Exclusion criteria

- First-year students with no clinical experience.
- Students who did not attend classes on days the data were collected.
- Students who did not voluntarily agree to participate in the study.

### Data collection instruments

#### Student descriptive information form

This form was developed by the researchers on the basis of the literature. The form includes nine questions about the students’ sociodemographic information [13, 14].

#### Barnett liking of children scale (BLCS)

Adapted for Turkish populations by Duyan and Gelbal, the Barnett Liking of Children Scale consists of 14 items scored between 1 (strongly disagree) and 7 (strongly agree). Higher scores indicate a greater liking of children. The internal consistency coefficient of the Turkish version is 0.92, and its test–retest reliability is 0.85 [15, 16]. In this study, the Cronbach’s alpha was 0.823, indicating high reliability.

#### Medication administration self-efficacy scale in children for nursing students (MASES-C)

Developed by Bektaş et al., the MASES-C consists of 16 items across two subdimensions: drug preparation (items 1–8) and drug administration (items 9–16). Items are rated on a five-point Likert scale, with scores ranging from 16 to 80; higher scores indicate greater self-efficacy in paediatric medication administration. The Cronbach’s alpha coefficients were reported as 0.94 for the entire scale, 0.91 for drug preparation and 0.87 for drug administration [17]. In our study, the Cronbach’s alpha coefficients were 0.948, 0.915, and 0.906, respectively.

#### Data collection

Before the data were collected, the purpose of the study was explained verbally and in writing to all the students who agreed to participate in the study, and their consent was obtained. Data were collected in the classroom environment through face-to-face interviews that lasted 10–15 min.

#### Evaluation of data

The data were analysed with IBM SPSS V23 and IBM AMOS V24. The conformity of the data to a normal distribution was examined using the Kolmogorov–Smirnov and Shapiro–Wilk tests, and the conformity to a normal distribution in path analysis was analysed using multiple normality assumptions. The Mann–Whitney U test was used to compare data that were not normally distributed between paired groups. The Kruskal–Wallis H test was used to compare data that did not conform to a normal distribution in groups of three or more, and multiple comparisons were analysed using Dunn’s test. Spearman’s rho correlation coefficient was used to examine the relationship between scale scores that did not fit the normal distribution. Path analysis was used to study the effects of the Liking of Children Scale on the subdimensions of drug preparation and drug administration. The results of the analysis are presented as the frequency (percentage) for categorical variables and as the mean ± standard deviation and median (minimum – maximum) for quantitative variables. The level of significance was set to *p* < 0.05.

#### Ethical considerations

Approval for the study was obtained from the Non-Interventional Clinical Research Ethics Committee of Van Yüzüncü Yıl University on September 12, 2022 (decision number 2022/12 − 07). Written consent was also obtained from the university where the study was conducted. All participants provided informed consent and voluntarily agreed to take part in the study. The study adhered to the principles of the Helsinki Declaration, scientific ethics and data confidentiality.

## Results

The mean age of the participants was 21.82 ± 1.94 years, and 68.8% were female. Other demographic characteristics, such as year of study, voluntary selection of nursing as a profession, interest in the child health and diseases nursing course, preferences for working in paediatric clinics and postgraduate plans are summarised in Table 1.

**Table 1:** Sociodemographic data

	Frequency	Percentage
Gender		
Female	212	68.8
Male	96	31.2
Year of Study		
Second year	80	26.0
Third year	121	39.3
Fourth year	107	34.7
Willingness to Choose Nursing as a Profession		
Yes	149	48.4
No	159	51.6
^1^ Interest in the Child Health and Diseases Nursing Course		
Yes	157	69.2
No	70	30.8
^1^ Enjoyment of Practising in Paediatric Clinics		
Yes	137	60.1
No	91	39.9
^1^ Nervousness about Practising in Paediatric Clinics		
Yes	156	68.4
No	72	31.6
Desire to Work in Paediatric Clinics After Graduation		
Yes	143	46.4
No	165	53.6
Intention to Pursue a Master’s Degree in Child Health		
Yes	145	47.1
No	163	52.9
Age	Mean ± S. Deviation	Mean ± S. Deviation
	21.82 ± 1.94	22 ± 19.36

^1^Because second-year students do not take the child health and diseases nursing course, the question ‘Do you like the child health and diseases course?’ was edited to be answered only by third- and fourth-year students; one student did not answer the question

**Table 2:** Descriptive statistics and correlation results of the scale scores and subdimensions

			Drug Preparation		Drug Administration		Self-efficacy Scale		Liking of Children Scale	
	Mean ± S. Deviation	Median (Min – Max)	*r*	*p*	*r*	*p*	*r*	*p*	*r*	*p*
Drug Preparation	25.79 ± 8.09	27 (8–40)	---	---						
Drug Administration	27.18 ± 7.9	27 (8–40)	0.804	**< 0.001**	---	---				
Self-efficacy Scale	52.97 ± 15.27	55 (16–80)	0.948	**< 0.001**	0.946	**< 0.001**	---	---		
Liking of Children Scale	66.65 ± 15.8	68 (14–98)	0.094	0.099	0.137	**0.016**	0.123	**0.031**	---	---

*r* = Spearman’s rho correlation coefficient

No statistically significant effect of the Liking of Children Scale was found on the drug preparation subdimension in nursing students (*p* > 0.05). A statistically significant effect of the Liking of Children Scale was found on the drug administration subdimension in nursing students (*p* < 0.05). A one-unit increase in the Liking of Children Scale resulted in a 0.068-unit increase in the drug administration subdimension (Table 2).

**Table 3:** Comparison of scale total scores and the subdimensions according to group

		Drug Preparation				Drug Administration				Self-efficacy Scale				Liking of Children Scale				
Gender																		
Female		24.73 ± 7.85		25 (8–40)		26.61 ± 7.9		27 (8–40)		51.34 ± 15.02			52 (16–80)	66.33 ± 15.83		68.5 (14–98)		
Male		28.14 ± 8.18		29 (8–40)		28.43 ± 7.78		28.5 (8–40)		56.56 ± 15.26			58 (16–80)	67.35 ± 15.8		68 (35–98)		
Test Statistic		7773				9021				8290.5				9887.5				
p*		**0.001**				0.11				**0.009**				0.69				
Year of Study																		
Second year		22.4 ± 7.56		22 (8–40)^c^		23.9 ± 7.71		24 (8–40)^b^		46.3 ± 14.6			46 (16–80)^b^	65.33 ± 15.77		68 (14–98)		
Third year		28.21 ± 8.46		29 (8–40)^b^		28.81 ± 8.29		30 (8–40)^a^		57.02 ± 16.2			60 (16–80)^a^	65.11 ± 13.12		68 (31–98)		
Fourth year		25.59 ± 7.12		27 (8–40)^a^		27.79 ± 6.84		27 (9–40)^a^		53.37 ± 12.9			55 (22–80)^a^	69.39 ± 18.21		72 (36–98)		
Test Statistic		27.779				21.740				27.354				0.169				
p**		**< 0.001**				**< 0.001**				**< 0.001**				0.155				
Willingness to Choose Nursing as a Profession																		
Yes		26.91 ± 7.74		28 (8–40)		28.12 ± 7.82		29 (8–40)		55.03 ± 14.82			57 (16–80)	68.49 ± 14.64		70 (14–98)		
No		24.74 ± 8.3		25 (8–40)		26.3 ± 7.88		27 (8–40)		51.03 ± 15.46			51 (16–80)	64.93 ± 16.68		68 (14–98)		
Test Statistic		10,071				10,316				10,067				10,339				
p*		**0.023**				0.050				**0.023**				0.053				
^1^ Interest in the Child Health and Diseases Nursing Course																		
Yes		27.79 ± 7.46		29 (8–40)		28.97 ± 7.54		30 (8–40)		56.76 ± 14.29	59 (16–80)			70.12 ± 14.75		71 (36–98)		
No		25.13 ± 8.79		26 (8–40)		26.91 ± 7.8		27 (8–40)		52.04 ± 15.69	49.5 (16–80)			60.46 ± 16.32		58.5 (31–98)		
Test Statistic		4411				4656				4468				3580				
p*		**0.018**				0.066				**0.025**				**< 0.001**				
^1^ Enjoyment of Practising in Paediatric Clinics																		
Yes		28.45 ± 7.69		30 (8–40)		28.91 ± 7.73		30 (8–40)		57.36 ± 14.77	59 (16–80)			69.94 ± 14.79		71 (36–98)		
No		24.76 ± 7.86		26 (8–40)		27.46 ± 7.48		28 (8–40)		52.22 ± 14.44	56 (16–80)			62.87 ± 16.45		64 (31–98)		
Test Statistic		4539				5588				4992				4638				
p*		**0.001**				0.185				**0.011**				**0.001**				
Desire to Work in Paediatric Clinics After Graduation																		
Yes		26.68 ± 8.38		28 (8–40)		27.73 ± 7.78		28 (8–40)		54.41 ± 15.73	56 (16–80)			70.6 ± 13.42		73 (36–98)		
No		25.02 ± 7.78		25 (8–40)		26.7 ± 7.98		27 (8–40)		51.72 ± 14.79	51 (16–80)			63.23 ± 16.92		63 (14–98)		
Test Statistic		10225.5				10947.5				10430.5				8427.5				
p*		**0.043**				0.275				0.079				**< 0.001**				
Intention to Pursue a Master’s Degree in Child Health																		
Yes		26.19 ± 8.53		27 (8–40)		27.39 ± 8.37		28 (8–40)		53.57 ± 16.33	55 (16–80)			70.23 ± 14.64		72 (36–98)		
No		25.44 ± 7.69		26 (8–40)		26.99 ± 7.47		27 (8–40)		52.43 ± 14.28	55 (16–80)			63.47 ± 16.16		65 (14–98)		
Test Statistic		11,029				11,355				11073.5				8935.5				
p*		0.312				0.553				0.340				**< 0.001**				
Age																		
*r*		0.127				0.182				0.160				0.132				
*p*		**0.026**				**0.001**				**0.005**				**0.020**				

*Mann–Whitney U test; **Kruskal–Wallis H test; ^a–c^: There is no difference between groups with the same letter; mean ± standard deviation; median (minimum – maximum). ^1^Because second-year students do not take the child health and diseases nursing course, the question ‘Do you like the child health and diseases course?’ was edited to be answered only by third- and fourth-year students. *r* = Spearman’s rho correlation coefficient

A statistically significant difference was observed between the drug preparation subdimension and variables such as gender, willingly choosing nursing, liking the child health course, enjoying working in paediatric clinics, and the desire to work in paediatric settings after graduation (*p* < 0.05). Significant differences were also found in both the drug preparation and administration subdimensions according to the student’s year of study (*p* < 0.001). With respect to the MASES-C total score, significant associations were found with gender, willingly having chosen nursing, liking the child health course, and preference for practice in paediatric clinics (*p* < 0.05). The Liking of Children Scale total score was significantly associated with enjoyment of working in paediatric clinics, interest in the child health course, and a desire to pursue a career or a master’s degree in paediatric health (*p* < 0.001). In addition, a strong positive correlation was observed between the drug preparation and administration subdimensions (*r* = 0.804; *p* < 0.001) and between the MASES-C total score and both the drug preparation (*r* = 0.948; *p* < 0.001) and drug administration subdimensions. Weak positive correlations were found between age and drug preparation (*r* = 0.127; *p* < 0.05), drug administration (*r* = 0.182; *p* < 0.05), MASES-C total score (*r* = 0.160; *p* < 0.05), and Liking of Children Scale total score (*r* = 0.132; *p* < 0.05). Although no significant correlation was detected between the Liking of Children Scale total score and the drug preparation subdimension, weak positive correlations were detected with the drug administration subdimension (*r* = 0.137; *p* < 0.05) and MASES-C total score (*r* = 0.123; *p* < 0.05) (Table 3).

**Table 4:** Path analysis results

Dependent Variable		Independent Variable	β^1^	β^2^	S. Error	Test Statistic	*p*	*R* ^2^
Drug Preparation	<---	Liking of Children Scale	0.039	0.075	0.029	1.324	0.185	0.006
Drug Administration	<---	Liking of Children Scale	0.068	0.137	0.028	2.425	**0.015**	0.019

β^1^: Unstandardised path coefficient; β^2^: Standardised path coefficient

There was no significant relationship between the Liking of Children Scale score and the drug preparation subdimension (*p* > 0.05), indicating that the level of liking of children does not influence this skill. However, there was a significant relationship between the Liking of Children Scale score and the drug administration subdimension (*p* < 0.05), suggesting that the level of liking of children may positively influence drug administration skills (Table 4).

**Fig. 1:**
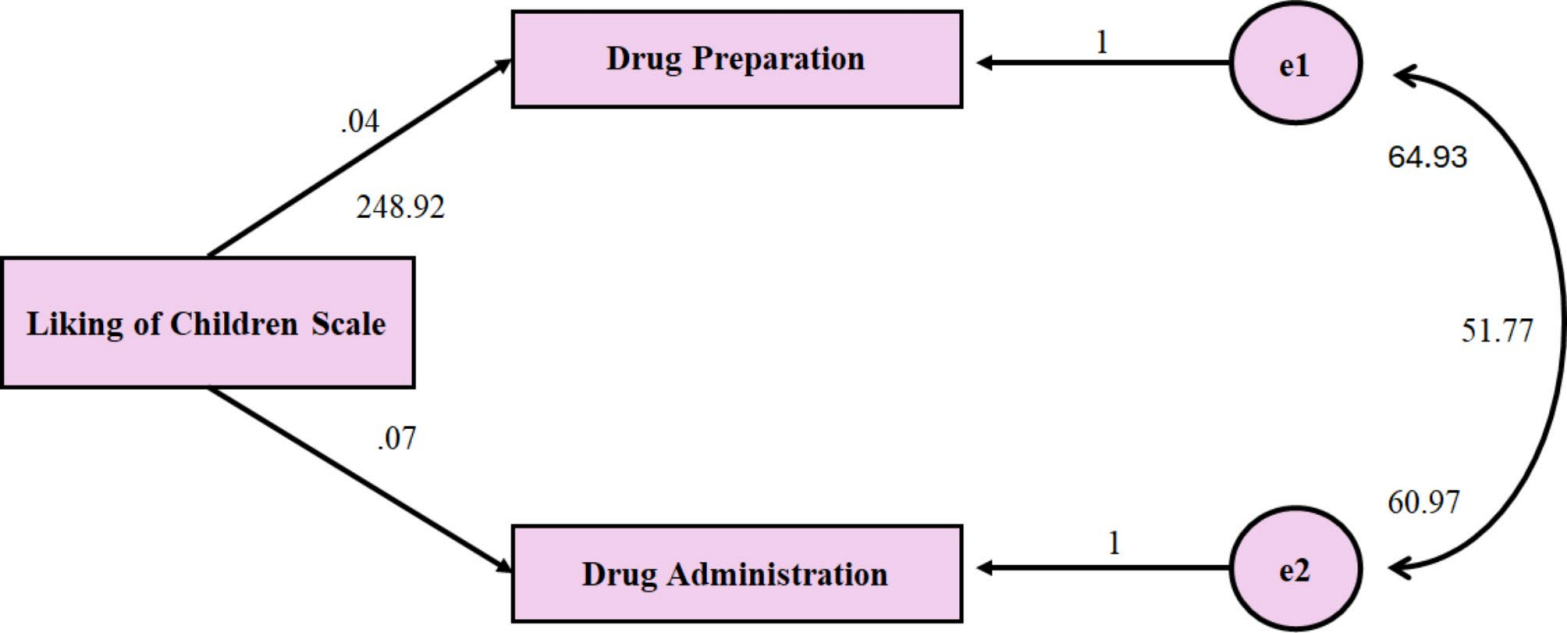
Unstandardised path coefficients

Figure 1 illustrates the path coefficients from the variable Liking of Children Scale to the subdimension of medication administration self-efficacy. The path coefficients indicate that an increase in the score on the Liking of Children Scale has a positive effect on medication administration self-efficacy (*p* < 0.05). This suggests that a positive emotional connection with children may enhance self-efficacy in the administration of medications. The model’s fit and the statistical significance of the coefficients support the study’s hypothesis (Fig. 1).

**Fig. 2:**
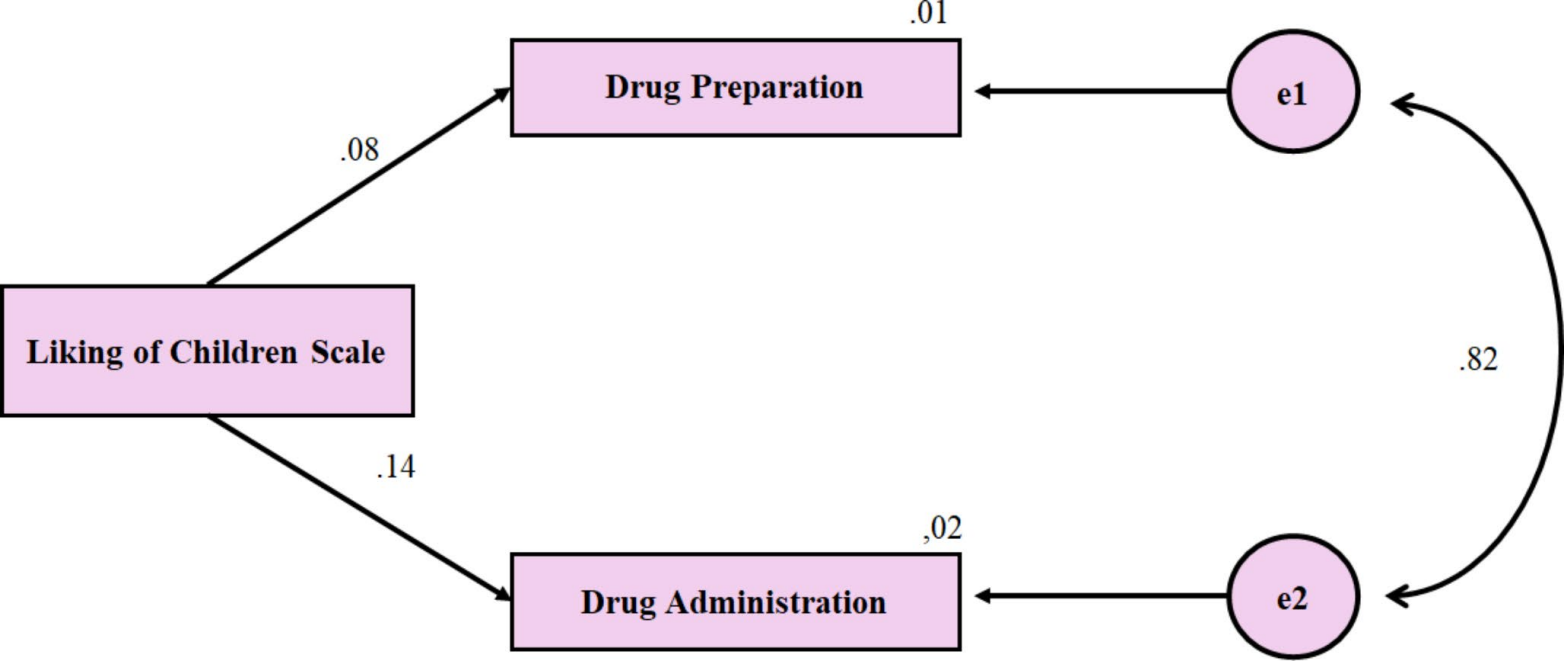
Standardised path coefficients

Figure 2 presents the standardised path coefficients. Standardised coefficients offer a clear comparison of the strength of relationships between variables. In this model, the relationship between the Liking of Children Scale and the medication administration subdimension is small but statistically significant (β = 0.137, *p* < 0.05). This result suggests that nursing students’ emotional inclinations towards children could influence their self-efficacy in paediatric medication administration (Fig. 2).

## Discussion

In our study, a positive but weak relationship was found between nursing students’ liking of children and their self-efficacy in paediatric medication administration. This significant positive relationship between the total score on the Liking of Children Scale and the total score on the Medication Administration Self-Efficacy Scale supports our hypothesis of a positive relationship between nursing students’ liking of children and their self-efficacy in administering medications to children. However, no study in the literature has reported a result exactly similar to this. Some studies closely related to our research have shown that emotional intelligence positively influences nurses’ clinical performance, job satisfaction and self-efficacy levels [18, 19]. Emotional intelligence has been shown to enhance both ethical decision-making processes and nurses’ self-efficacy in clinical practice [20]. In particular, nurses with high emotional intelligence are successful in engaging in empathic interactions with children, and these interactions are reported to reduce children’s anxiety and stress levels, contributing to good treatment outcomes [21]. The impact of emotional intelligence is not limited to individual performance; it also positively influences nurses’ job satisfaction and self-efficacy in clinical practice. A supportive work environment promotes the development of emotional intelligence and strengthens the sense of self-efficacy, thereby improving the quality of paediatric care [22]. Conversely, a lack of such support can lead to burnout and job dissatisfaction, weakening nurses’ clinical performance [18, 23].

It has been noted that male students, despite facing challenges as a minority in a female-dominated profession, also have more opportunities for professional advancement [24]. In this study, male nursing students performed better than their female counterparts in preparing medications for children. Similarly, a study that examined nursing students’ ability to correctly calculate medication dosages reported that male students scored higher in all areas of a mathematics test [25]. These findings suggest that male students’ stronger mathematical skills may contribute to their success in precise and technical tasks, such as medication preparation. It has also been emphasised that male students feel the need to prove themselves not only in clinical skills but also in areas such as strength, leadership and technical competencies [26, 27]. As a consequence, their higher scores in stereotypically masculine traits, such as dominance and achievement, have been suggested to positively influence their confidence and assertiveness in medication administration processes [28]. These results help explain the stronger performance of male students in medication preparation and provide an important perspective on the impact of gender on clinical performance. In conclusion, further studies are needed to explore the broad effects of gender on nursing students’ clinical skills.

Although the majority of nursing students can successfully perform simple drug calculations, many other students have difficulty with more complex calculations and calculations that require conceptual understanding [29]. The performance of educators in this field can significantly affect student nurses’ pharmacological knowledge and medication management skills [30]. Furthermore, the supervision process is critical in guiding learners to adhere to best practices and prevent medication errors [31]. In this context, nursing education that focuses on pharmacology, drug calculation skills and effective teaching practices is critical if students are to gain the competence needed to prepare drugs correctly and safely. In our study, the highest value was observed in the third-year students in terms of drug preparation and administration self-efficacy. It is thought that the third-year paediatric nursing course is responsible for this result. Taking the third-year paediatric nursing course and the integrated clinical practices associated with it may have played a critical role in enhancing students’ self-efficacy in medication preparation and administration. During this year, students learn the specific requirements of paediatric nursing and apply their pharmacological knowledge and medication management skills in a practical setting. The literature suggests that such clinical experiences and specialised courses significantly increase students’ confidence and clinical skills [30]. Increased exposure to hands-on experiences and practical knowledge in the third academic year likely contributed to the improved performance of students in medication preparation processes.

In our study, students who willingly chose nursing as a profession had higher drug preparation self-efficacy than did those who chose it unwillingly. Consideration of the effects of self-efficacy on nursing students’ performance and attitudes is essential if the relationship between nursing students’ choice of profession and their drug preparation self-efficacy is to be addressed [32]. Self-efficacy plays an important role in shaping nursing students’ clinical performance, communication skills and general competence [33–35]. Moreover, career choice and self-efficacy are intertwined with students’ satisfaction with the clinical learning environment and readiness for practice [9]. Nursing students who feel they are suited to the nursing profession show better general self-efficacy than those who do not feel suited to the profession [32].

In our study, students who liked the child health and diseases nursing course and practice had greater self-efficacy in drug administration and liking of children than did those who did not. The satisfaction and fulfilment experienced by paediatric nurses are associated with high levels of self-efficacy, emotional involvement and positive attitudes towards families. These factors ultimately contribute to successful outcomes in child care tasks [8, 36–38]. Furthermore, the relationship between self-efficacy perceived by paediatric nurses and the quality of paediatric nursing care has been emphasised, and it has been suggested that higher self-efficacy may lead to better professional performance [39]. It has also been shown that educational programmes can increase student nurses’ self-efficacy in managing paediatric pain and that targeted education can have a positive effect on self-efficacy in specific nursing tasks [40]. Individuals who like paediatric nursing have greater self-efficacy in tasks related to childcare. This suggests that liking paediatric courses and practices may increase self-efficacy in tasks such as drug management and encourage the liking of children among nursing students.

Our study found that students who wanted to work in paediatric clinics after graduation and/or obtain a master’s degree in paediatrics had a higher liking of children than those who did not. Nursing students who wanted to work in paediatric clinics showed a desire to play with children, which indicates a positive attitude towards children [41]. It has been reported that the desire to work in paediatric clinics is positively related to the liking of children in paediatric nursing students [42]. An analysis of the liking of children and the parenting attitudes of nurses working in paediatric clinics has revealed a correlation between working in a paediatric environment and attitudes towards children [43]. In conclusion, the literature supports the view that individuals who want to work in paediatric clinics after graduation are more likely to like children than those who do not express such a career preference.

In our study, as the students’ age increased, their self-efficacy in administering medications to children and their affection for children also increased. However, it is believed that this increase is related not only to age but rather to the growing clinical experience that comes with age, which may be a more significant factor. Emotional intelligence has been shown to increase with age, and this is related to greater self-efficacy as nursing students gain more clinical experience [44]. In addition, older students report greater competence in nursing care as their clinical experience increases, which in turn contributes to greater self-efficacy in tasks such as medication management [45]. Moreover, as nursing students gain clinical experience, they feel more comfortable, their anxiety levels decrease, and their perceptions of self-efficacy improve [42]. In conclusion, the literature supports a positive relationship between nursing students’ age and their self-efficacy in clinical tasks and affection for children, driven primarily by their increasing clinical experiences. As students gain more experience, they are likely to develop greater self-efficacy in medication management and show greater interest in working with children.

Drug administration is a process that involves administration, documentation, management and monitoring; these tasks occupy approximately 40% of nurses’ time [46]. The most common problems in paediatric patients are drug administration errors and incorrect intravenous drug infusions [47]. Competence in drug management is positively correlated with nurses’ general nursing skills [48]. One study analysed nursing students’ drug dose calculation skills and suggested that their competence in drug-related tasks could be improved through interventions and education [49]. Peer support systems may positively affect student nurses’ drug management skills by increasing their knowledge and confidence [50]. Another study, which compared the numerical skills and drug calculation abilities of nursing students and working nurses, emphasised the importance of competence in drug calculations for safe drug administration [51]. In this context, the literature provides an important perspective on the importance of general nursing skills, educational interventions, peer support and numerical abilities in influencing nursing students’ competence in medication-related tasks, although the relationship between the liking of children and drug administration skills has not been clearly demonstrated [48–51]. In our study, a statistically significant positive and very weak correlation was found between the liking of children and drug administration. This finding shows that emotional factors may also be effective in the process of developing nursing students’ competencies in drug-related tasks.

These findings highlight the importance of considering emotional factors, such as the liking of children, in nursing education. Emotional and psychological components may play a significant role in shaping nursing students’ self-efficacy, in particular in paediatric care contexts. The incorporation of these elements into nursing curricula could increase students’ motivation and commitment, ultimately improving their clinical skills, including drug administration. Given the lack of similar findings in the literature, further research is needed to explore the broad impact of emotional factors on clinical performance. Future studies could address this gap by examining the influence of emotional and psychological aspects on nursing students’ development of clinical competencies, providing valuable insights for enhancing nursing education programmes.

## Conclusions and recommendations

This study demonstrated a positive relationship between nursing students’ affection for children and their self-efficacy in paediatric medication administration. The results indicate that students’ emotional characteristics significantly influence their clinical performance and suggest that enhancing the content of nursing education that focuses on developing emotional intelligence and empathy skills is important. Students should also be encouraged to gain more experience in paediatric clinical settings, and further research is needed to explore the reasons behind male students’ success in medication preparation. Future studies in these areas can provide in-depth insights and contribute to the improvement of nursing education.

### Strengths

This study is one of the first to examine the relationship between nursing students’ self-efficacy in paediatric medication administration and their affection for children. Although emotional intelligence has been previously studied, the inclusion of affection for children in this context is a unique contribution of this research. The validity of the scales used is high, which enhances the reliability of the data. Addressing gender differences also provides a new perspective in this area.

### Limitations

The study was conducted at only one university, which may limit the generalisability of the findings. Furthermore, it was conducted within a specific time frame, so long-term effects were not evaluated.

## Data Availability

No datasets were generated or analysed during the current study.
